# One hundred and seventy-six cases of adult tinea capitis: a 14-year retrospective, single-center study in *Hangzhou*, China

**DOI:** 10.3389/fmicb.2026.1862479

**Published:** 2026-07-03

**Authors:** Hui-Lin Zhi, Xiu-Jiao Xia, Ze-Hu Liu

**Affiliations:** Department of Dermatology, Hangzhou Third People’s Hospital, Affiliated Zhejiang Chinese Medicine University, Hangzhou, China

**Keywords:** adult tinea capitis, dermatophyte, epidemiology, female, *Microsporum canis*, *Trichophyton violaceum*

## Abstract

**Introduction:**

Tinea capitis is a dermatophyte infection of the scalp and hair that predominantly affects preschool children; however, adult cases have received increasing attention in recent years. This study aims to evaluate the epidemiological characteristics of adult tinea capitis in *Hangzhou*, eastern China.

**Materials and methods:**

The epidemiological data of adult tinea capitis patients referred to the mycology laboratory of *Hangzhou Third People’s Hospital* from 2012 to 2025 were evaluated retrospectively. The epidemiological profiles were collected and analyzed, mainly including the pathogen spectrum and the present potential risk factors.

**Results:**

A total of 176 adult tinea capitis were included. The sex ratio was 1:2.59, with 49 (27.8%) males and 127 (72.2%) females. Women over 45 years of age accounted for 102 cases (58%). A total of 130 strains (82.8%) were isolated from 157 adult tinea capitis cases that had fungal culture performed. Cases with negative cultures were excluded from pathogen analysis. The most common pathogens were *Trichophyton violaceum* (36 cases, 27.7%) and *Microsporum canis* (36 cases, 27.7%), followed by *Trichophyton rubrum* (29 cases, 22.3%). Anthropophilic dermatophytes accounted for 80 strains (61.5%). Notably, 59 patients (33.5%) had concurrent dermatophyte infections at other body sites.

**Conclusion:**

This study indicates that adult tinea capitis patients were predominantly female, especially those over 45 years of age. The predominant dermatophyte were *T. violaceum* and *M. canis*, followed by *T. rubrum*. This study provides a scientific basis for the prevention and control of ATC in the *Hangzhou* region.

## 1 Introduction

Tinea capitis, a dermatophyte infection that primarily affects the scalp, has long been considered a condition predominantly seen in children (Gupta et al., 2024). However, emerging evidence suggests that adult tinea capitis is an under-recognized yet significant clinical entity, posing distinct challenges and implications (Auchus et al., 2016; Hill et al., 2024). Adult tinea capitis (ATC), although less prevalent, its atypical presentation may lead to misdiagnosis as other inflammatory dermatoses, such as seborrheic dermatitis, eczema and folliculitis (Liang et al., 2020). Consequently, ATCs are frequently disregarded, resulting in protracted disease courses and significant hair loss, which substantially impacts on both mental and physical health (Segal-Engelchin and Shvarts, 2020). It has been reported that ATC was particularly affecting postmenopausal women and immunocompromised patients (Khosravi et al., 2016). However, it has also been reported to be common in healthy individuals (Liang et al., 2020). The distribution of dermatophyte species in ATC varies due to differences in geographicy and lifestyle (Leung et al., 2020). Epidemiological data on ATC in eastern China remain limited. Therefore, this study aimed to evaluate the epidemiology and fungal characteristics of ATC in *Hangzhou*, eastern China, over the past 14 years.

## 2 Materials and methods

### 2.1 Study population and clinical data

This study retrospectively collected clinical data of ATC patients diagnosed by the Mycology Laboratory of the Third People’s Hospital of Hangzhou from January 2012 to December 2025. The demographic data collected included the patient’s age, gender, and mycology examination results. Relevant influencing factors such as fungal infections in other sites, history of pet contact, and immunocompromised diseases were also recorded. The inclusion criteria for the study were as follows: (1) must be over 18 years old; and (2) the presence of clinical symptoms and microscopic true hair invasion in the form of ectothrix or endothrix; or (3) the presence of clinical symptoms and culture-proven dermatophyte infection of the scalp and hair.

### 2.2 Direct examination and culture

Specimens were collected on slides and examined directly using 10% potassium hydroxide (KOH) or calcofluor white staining to observe details of hyphae and spores. The samples were then cultured in Sabouraud dextrose agar slants supplemented with chloramphenicol and incubated at 25 °C for 10 to 15 days. The cultures were monitored daily for the presence of colony-forming units. For culture tubes showed no growth after 15 days, incubation was continued for an additional 3-4 weeks; if no growth was observed by that time, the culture was reported as negative.

### 2.3 Identification of fungal isolates

Fungal strains are preliminarily identified using optical microscopy in combination with macro-characteristics. Macroscopic identification involved evaluating the texture, topography, margins, colors, obverse and reverse pigments, and growth time of the colonies. Microscopic identification was carried out by sub-culturing the fungus on potato agar using a slide culture technique. For species that cannot be identified based on morphology, we amplified and sequenced the internal transcribed spacer (ITS) region using the primers ITS1 (5′-TCCGTAGGTGAACCTGCGG-3′) and ITS4 (5′- TCCTCCGCTTATTGATATGC-3′). The resulting sequences were assembled and searched against the NCBI database. We are unable to distinguish between *T. mentagrophytes* and *T. interdigitale* using either morphology or ITS sequencing methods, so we classify them as the “*T. mentagrophytes complex*.”

### 2.4 Statistical analysis

All statistical analyses were performed using R software (version 4.2.2). Categorical data were compared using the Chi-square test or Fisher’s exact test, *p* < 0.05 was considered statistically significant. The Cochran-Armitage test for trend was used to evaluate the linear trend of proportions across ordered age groups (18–44, 45–69, 70–96 years). Odds ratios (ORs) with 95% confidence intervals (CIs) were calculated using the unconditional maximum likelihood method.

## 3 Results

### 3.1 Distribution of ATC by age and sex

In this study, a total of 176 patients were diagnosed with ATC over the 14-year period (2012–2025). ATC patients included 49 males (27.8%) and 127 females (72.2%), with a sex ratio of 1:2.59. The age group 45–69 years had the highest number of infections (68 cases, 38.6%), followed by the 70–96 years group (66 cases, 37.5%), and the 18–44 years group (42 cases, 23.9%) was the lowest. Notably, ATC was more common in females aged >45 years, accounting for 102 of the 176 total cases (58%) (Table 1).

**Table 1:** Comparison of age and gender in adult tinea capitis among four age groups.

Age (years)	18–44	45–69	70–96	*N* (%)
Male	17	18	14	49 (27.8%)
Female	25	50	52	127 (72.2%)
Total	42 (23.9%)	68 (38.6%)	66 (37.5%)	176 (100%)

### 3.2 ATC pathogen distribution by age group

Among the 176 total cases, 19 patients had positive direct microscopy but did not undergo fungal culture at the time. Fungal cultures were conducted on the remaining 157 patients. A total of 130 strains of fungi were successfully isolated from 157 ATC. The culture positivity rate was 82.8%. Of these, 80 strains (61%) were anthropophilic dermatophytes. The proportion of anthropophilic dermatophytes increased across the three age groups (35.7, 64.0, and 86.8%, respectively) (*p* < 0.001). The most frequently identified anthropophilic dermatophytes was *T. violaceum* (36 strains, 27.7%), followed by *T. rubrum* (29 strains, 22.3%). Other pathogens included *T. tonsurans* (14 strains, 10.8%) and *T. schoenleinii* (1 strain, 0.7%). Zoophilic dermatophytes accounted for 48 strains (36.9%). The most frequently identified zoophilic dermatophytes was *M. canis* (36 strains, 27.7%), followed by *T. mentagrophytes complex* (12 strains, 9.2%). In addition, the geophilic dermatophytes *Nannizzia gypsea* was identified in only 2 cases (1.5%). Notably, *T. rubrum* predominantly affected middle-aged and elderly patients, with the highest proportion in the 70–96 years age group (Cochran-Armitage test: *Z* = 5.94, *p* < 0.001). However, *M. canis* predominantly affected young adults, with the highest proportion in the 18–44 years age group (*p* < 0.05) (Table 2).

**Table 2:** Fungal species distribution of adult tinea capitis cases across different age groups.

Species, no. (%)	18–44	45–69	70–96	*N* = 130 (100%)
Anthropophilic	15	32	33	80 (61.5%)
*T. violaceum*	12	15	9	36 (27.7%)
*T. rubrum*	0	11	18	29 (22.3%)
*T. tonsurans*	3	6	5	14 (10.8%)
*T. schoenleinii*	0	0	1	1 (0.7%)
Zoophilic	26	17	5	48 (36.9%)
*M. canis*	19	12	5	36 (27.7%)
*T. mentagrophytes complex*	7	5	0	12 (9.2%)
Geophilic	1	1	0	2 (1.5%)
*N. gypsea*	1	1	0	2 (1.5%)
Total	42	50	38	130

### 3.3 ATC pathogen distribution by time period

The distribution of fungal species in ATC cases was analyzed across different time periods, and changes in the number of infections and pathogen species were assessed using 7-year intervals. The results showed 57 cases (32.4%) of ATC between 2012 and 2018, and 119 cases (67.6%) between 2019 and 2025. In both periods, anthropophilic dermatophytes remained the predominant pathogens. However, the distribution of pathogen species differed between the two time periods. From 2012 to 2018, the most common pathogen was *T. violaceum* (26.3%), followed by *M. canis* (22.8%) and the *T. mentagrophytes complex* (15.7%). From 2019 to 2025, the most common pathogen was *T. rubrum* (20.1%), followed by *M. canis* (19.3%) and *T. violaceum* (17.6%). The difference in fungal species composition between the two time periods was highly significant (*p* < 0.001) (Table 3).

**Table 3:** Fungal species distribution of adult tinea capitis cases across different time periods.

Species, no. (%)	2012–2018	2019–2025
Total	57	119
Culture positive	48	82
*T. violaceum*	15 (26.3%)	21 (17.6%)
*T. rubrum*	5 (8.7%)	24 (20.1%)
*T. tonsurans*	5 (8.7%)	9 (7.5%)
*T. schoenleinii*	0 (0.0%)	1 (0.8%)
*M. canis*	13 (22.8%)	23 (19.3%)
*T. mentagrophytes complex*	9 (15.7%)	3 (2.5%)
*N. gypsea*	1 (1.7%)	1 (0.8%)

### 3.4 Risk factors and clinical manifestations of ATC

The clinical data revealed that 59 patients (33.5%) had concurrent dermatophyte infections at other body sites. The most frequently involved sites were the hands and fingernails (18 cases), followed by the face and neck (15 cases). There were 10 cases of diffuse tinea corporis, 8 cases of tinea cruris, and 3 cases of localized tinea corporis. Of the 59 patients with concurrent infections, a total of 32 cases of ATC were caused by *T. rubrum* and *T. violaceum*. Among these, ATC caused by *T. rubrum* with multi-site infections was 14 cases. Among these 14 cases, 5 patients (35.7%) underwent fungal culture of the concomitant tinea lesions at other body sites, and *T. rubrum* was isolated from those sites. The accompanying sites in 9 of these cases (64.3%) were the fingernails and hands. Among the ATC cases caused by *T. violaceum* with multi-site infections (12 cases), the accompanying sites included the face (6 cases, 50%), as well as the hands and nails (3 cases), See the Supplementary materials for details.

Four patients (2.3%) had concomitant immunocompromising diseases. These included diabetes mellitus (1 cases), pemphigus (1 cases), thyroid cancer (1 case), and lung cancer (1 case). Additionally, 9 patients (5.1%) had a definite history of contacted with animals, specifically 5 patients had contact with cat, 2 patient had contact with dog, and 2 patients had contact with cat and dog (Table 4).

**Table 4:** Possible mode of contributing factors in confirmed cases of adult tinea capitis.

Relevant risk factors	Number (*n*)	Percentage (%)
Other parts of tinea	59	33.5%
Hands and fingernails	18	
Face and neck	15	
Diffuse tinea corporis	10	
Truncus	8	
Cruris	3	
Other	5	
Concomitant diseases	4	2.3%
Diabetes mellitus	1	
Pemphigus	1	
Thyroid cancer	1	
Lung cancer	1	
Contact with animals	9	5.1%
Cats	5	
Dogs	2	
Cats and dogs	2	

**Figure 1:**
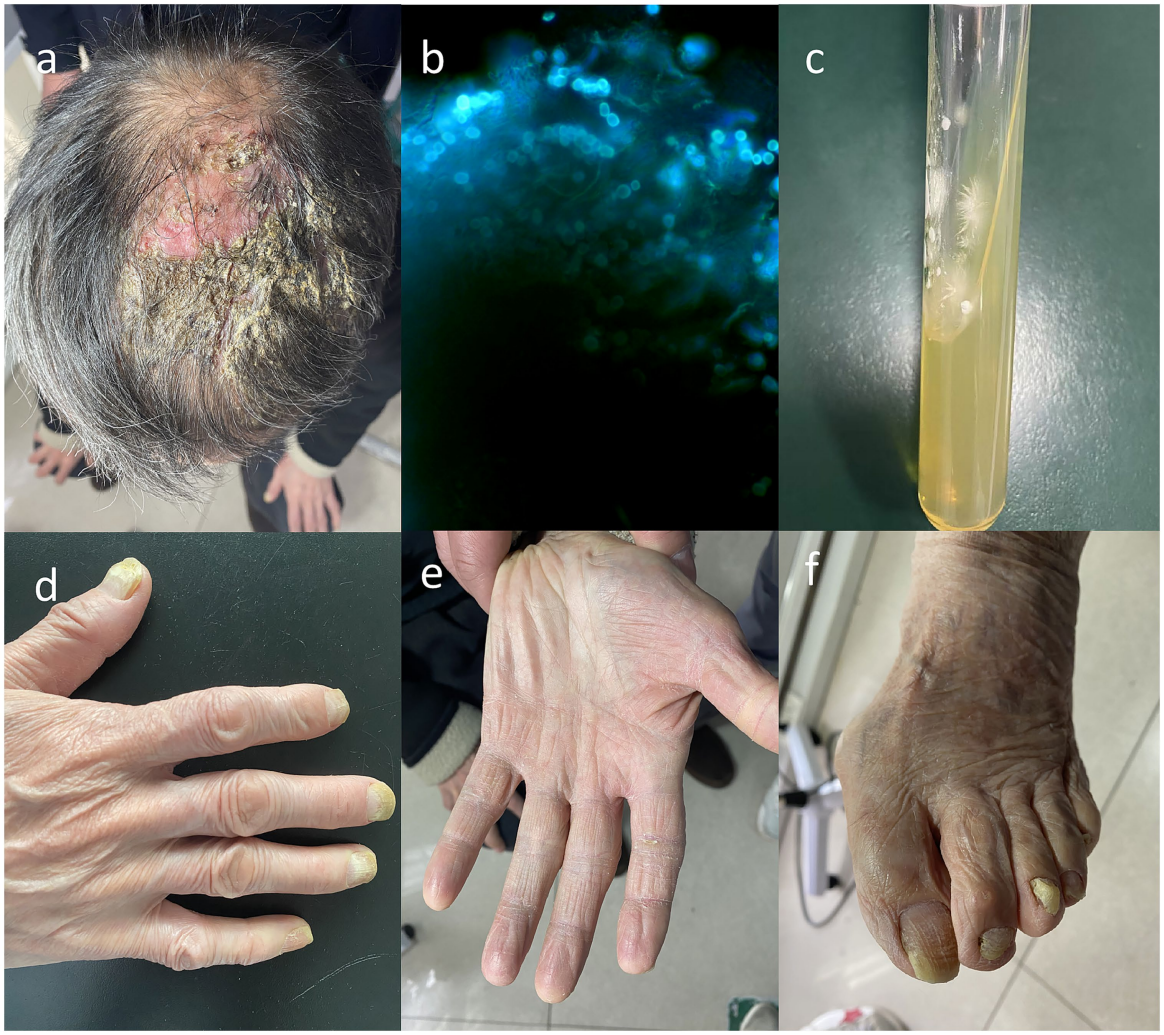
An 81-year-old male presented with clinical indications of showing erythema, scaling, and crust formation on the scalp **(a)**. Direct examination with calcofluor white staining a large number of endothrix spores (**b**, 400×). After 10 days of fungal culture, *T. rubrum* colonies were observed **(c)**. Tinea manuum and onychomycosis effected by *T. rubrum* **(d–f)**.

**Figure 2:**
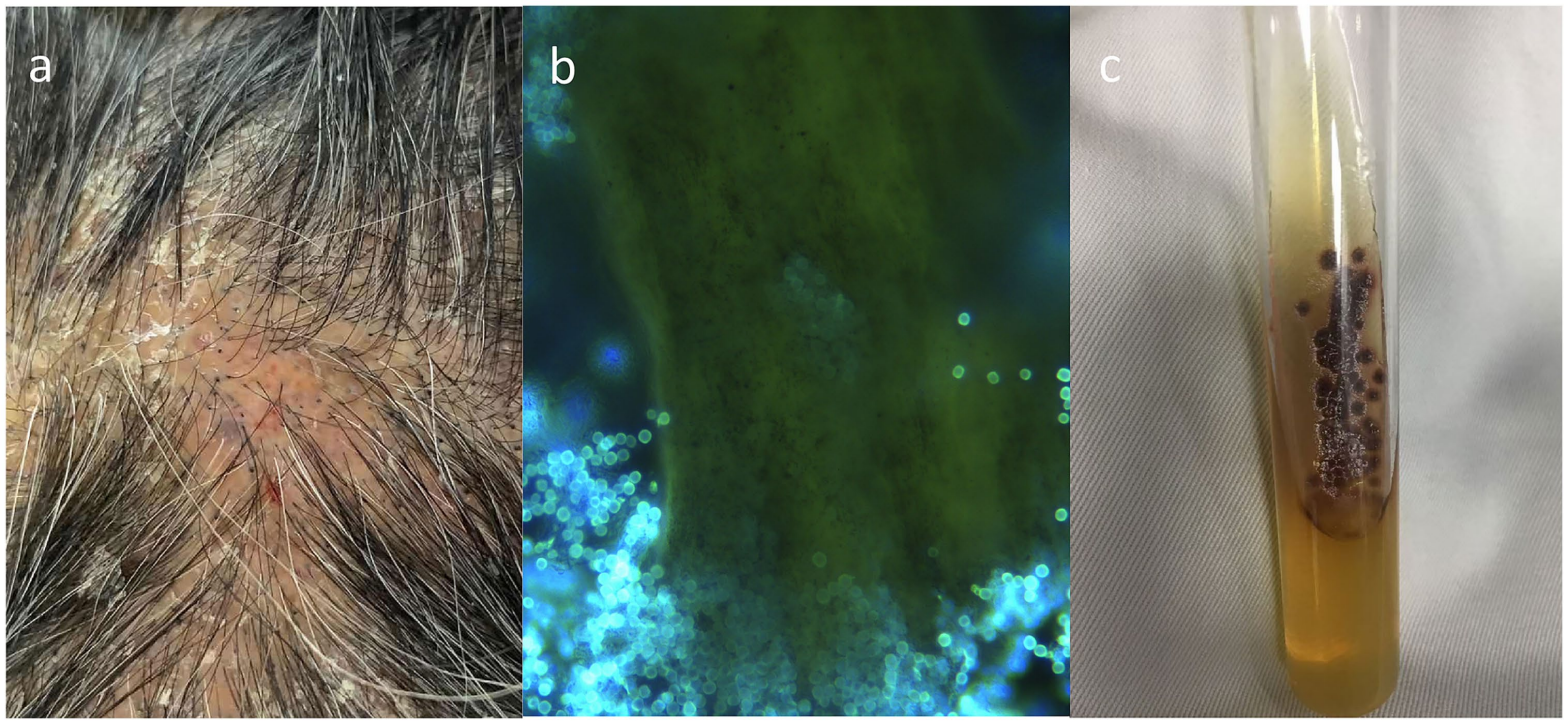
A 67-year-old female patient presented with scalp alopecia and black dots **(a)**. Direct examination with calcofluor white staining showed endothrix spores, the arthroconidia seen on the exterior of the hair were an artifact of pressure applied during microscopic slide preparation, which forced the internal spores outward. (**b**, 400×). Fungal culture revealed *T. violaceum* **(c)***.*

**Figure 3:**
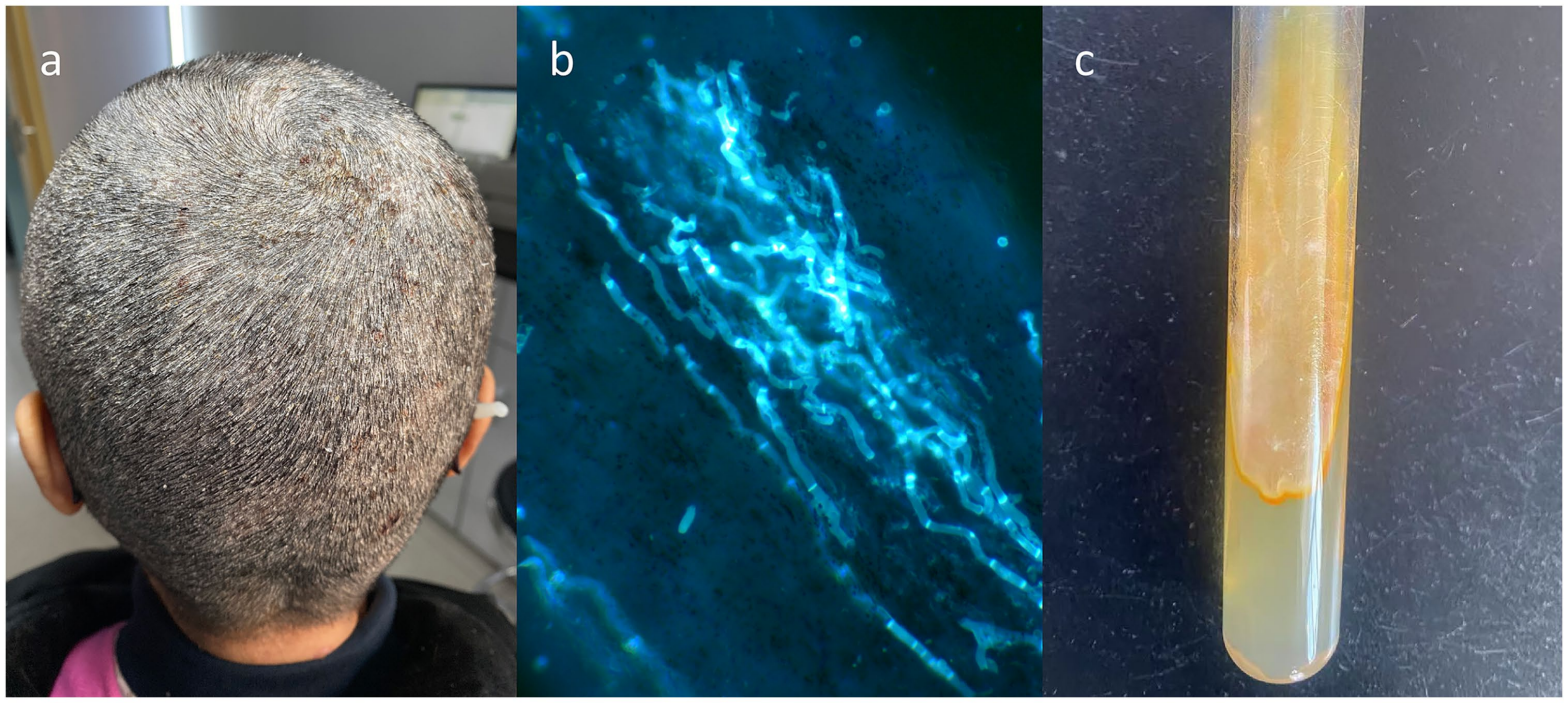
A 66-year-old female patient with lung cancer undergoing chemotherapy presented with diffuse white scaling and scattered pustules across the scalp **(a)**. Direct examination with calcofluor white staining showed ectothrix hyphae (**b**, 400×). Fungal culture revealed *M. canis* **(c)**.

Regarding clinical features, ATC presented with atypical and variable manifestations. The diffuse scale (seborrheic dermatitis-like) variant was a common clinical presentation (Figure 1) (El-Khalawany et al., 2013), followed by black dot (Figure 2), gray patch (Figure 3), Pustular type (Park et al., 2019) and kerion.

## 4 Discussion

Tinea capitis remains a significant global public health concern. Although ATC has historically been considered uncommon in adults, recent reports suggest an increasing trend in prevalence (Leung et al., 2020). This shift is believed to be influenced by various demographic factors, including age, gender, and immune status (Khosravi et al., 2016). Understanding these epidemiological trends is essential for developing targeted public health strategies and for recognizing that conditions once considered pediatric issues are also relevant to adults.

The demographic dynamics of ATC are multifaceted and vary across populations. Age and gender have emerged as significant determinants in the epidemiology of this condition. This study showed that ATC was more prevalent among women over the age of 45 (58%). This is consistent with the results of a Korean study, which showed a higher incidence in postmenopausal women (Khosravi et al., 2016). This observation may be attributed to the reduction in sebaceous gland secretion associated with decreased blood estrogen levels during menopause (Segal-Engelchin and Shvarts, 2020). However, other studies have reported differing patterns: ATC predominantly affects young women in Dakar, Senegal (Diop et al., 2019), and primarily affects males in Hubei, China (He et al., 2021).

The pathogens responsible for ATC may vary according to geographical location, climate and time (Chen et al., 2021; Miao et al., 2026). In France, *T. tonsurans* was the predominant dermatophyte (Gits-Muselli et al., 2017), whereas in Italy and Portugal, *T. rubrum* was the most common dermatophyte (Calabro et al., 2015; Duarte et al., 2019). *T. violaceum* was found to be prevalent in Egypt and Tunisia (Mebazaa et al., 2010; El-Khalawany et al., 2013). *T. soudanense* (57.6%) was the most common species in Dakar, Senegal (Diongue et al., 2025). In China, the distribution of pathogens varies substantially by geography and climate. *Trichophyton* species, particularly *T. violaceum*, have been identified as the main pathogens in adult populations in various studies, including in *Wuhan* (Dong et al., 2023) and *Jiangxi* (126 cases, 75%) (Jin et al., 2023). However, *M. canis* (40%) was the main pathogen in *Guangxi* from January 2019 to July 2022 (Zheng et al., 2023) and *T. rubrum* was the most common species in China reported by Liang et al. (2020). This study found that *T. violaceum* and *M. canis* were the most common dermatophytes in ATC. In addition, the anthropophilic dermatophytes affected adults significantly more than children in our center (Zhi et al., 2021). These results emphasize the importance of conducting ATC research in different regions and at different times.

In this study, the number of ATC cases between 2019 and 2025 was approximately 2.1 times that observed between 2012 and 2018. This increase may be attributable to the introduction of fluorescence staining microscopy in our laboratory after 2016, which enhanced the detection rate of fungal-infected hair. Furthermore, the distribution of dermatophyte species changed over time. Specifically, the pathogenic fungi shifted from *T. violaceum*, *M. canis*, and the *T. mentagrophytes complex* to *T. rubrum*, *T. violaceum*, and *M. canis*. This shift may be explained by the fact that scalp infection caused by *T. rubrum* often presents as a diffuse scaling (seborrheic dermatitis-like) variant, which is relatively mild and therefore overlooked (Liang et al., 2026). With the advancement of fungal detection techniques and the improvement of physicians’ diagnostic skills, the detection rate of this pathogen has increased.

The propensity of adults to contract tinea capitis is influenced by a range of host factors, including immune status and lifestyle practices (Khosravi et al., 2016). We analyzed risk factors associated with the sources of anthropophilic ATC pathogens. Among the ATC cases caused by *T. rubrum* with multi-site infections, 35.7% also had the pathogen isolated from other body sites. The most common accompanying sites were the hands and fingernails (64.3%). This might suggest that scalp infection is caused by scratching in patients with tinea manuum or tinea unguium (Park et al., 2019). Among the ATC cases caused by *T. violaceum* with multi-site infections, the face was the most common accompanying site (50%), suggesting direct spread between the scalp and the face as a transmission route for this pathogen. Additionally, *T. rubrum* predominantly affected elderly patients, whereas *M. canis* predominantly affected young adults. Therefore, elderly individuals should maintain hand hygiene and scalp cleanliness, young people should avoid contact with animals, and timely diagnosis and treatment may help reduce ATC infections. In fact, ATC can infect both healthy individuals and immuno-suppressed patients. In this study, only four patients (2.3%) had immunocompromising conditions, which is consistent with previous some reports (Liang et al., 2020). Consequently, clinicians should consider tinea capitis as a differential diagnosis for scalp diseases even in healthy individuals.

ATC was mainly caused by the anthropophilic dermatophytes and differs from infection in children, which was mainly caused by the zoophilic dermatophytes (Zhi et al., 2021). Due to inhibition by sebum, *Microsporum* is less likely to remain on the scalp after puberty, whereas *Trichophyton* can persist on the scalp of teenagers and adults for years (Chen et al., 2021). Moreover, *T. violaceum* is the most common dermatophyte identified in asymptomatic tinea capitis carriers (Aharaz et al., 2021). All these findings may explain why the anthropophilic dermatophyte was the most common pathogens of ATC in *Hangzhou*, China.

This study has the following limitations: As a single-center retrospective study, the data may be subject to regional biases and incomplete data collection, Other limitations include the absence of a control/comparison group, the lack of statistical modeling of risk factors, and the inability to distinguish members of the *T. mentagrophytes complex.* Further multi-center prospective studies focusing on ATC are needed to reduce potential biases and to provide more in-depth research in this area.

## 5 Conclusion

ATC is primarily caused by anthropophilic dermatophytes and is more prevalent in women, particularly postmenopausal women. The predominant dermatophytes causing ATC were *T. violaceum* and *M. canis*, followed by *T. rubrum*. This study provides a scientific basis regarding the predominant circulating pathogens of ATC in the *Hangzhou* region and offers insights for further prevention and control efforts.

## Data Availability

The original contributions presented in the study are included in the article/Supplementary material, further inquiries can be directed to the corresponding authors.
