# Supplementary material for: One hundred and seventy-six cases of adult tinea capitis: a 14-year retrospective, single-center study in Hangzhou, China
Source: Front Microbiol. 2026 Jul 3;17:1862479. doi: 10.3389/fmicb.2026.1862479 (PMC13376864; doi:10.3389/fmicb.2026.1862479)
Supplement: Supplementary file 1 [file Table_1.DOCX]

Table 1 Cases of ATC caused by *T. rubrum* with concomitant tinea at other body sites

| **No.** | **Gender** | **Age** | **Positive direct microscopy (site)** | **Positive culture (species)** |
| --- | --- | --- | --- | --- |
| 1 | Male | 67 | Tinea corporis | Not done |
| 2 | Female | 71 | Nail，fingernail | Not done |
| 3 | Male | 72 | Nail, tinea cruris | Not done |
| 4 | Female | 62 | Hand, face | Not done |
| 5 | Female | 66 | Hand, face | Not done |
| 6 | Male | 76 | Nailzu | Not done |
| 7 | Female | 60 | Toenail | *T. rubrum* |
| 8 | Male | 65 | Hand, toenail | *T. rubrum* |
| 9 | Male | 79 | Face | *T. rubrum* |
| 10 | Male | 81 | Nail, toenail, hand and foot | *T. rubrum* |
| 11 | Female | 66 | Nail | Not done |
| 12 | Female | 84 | Buttock | *T. rubrum* |
| 13 | Male | 64 | Face | Not done |
| 14 | Male | 69 | Hand | Not done |

Table 2 Cases of ATC caused by *T. violaceum* with concomitant tinea at other body sites

| **No.** | **Gender** | **Age** | Positive direct microscopy (site) |
| --- | --- | --- | --- |
| 1 | Female | 20 | Hand |
| 2 | Male | 18 | Back |
| 3 | Female | 19 | Face |
| 4 | Female | 32 | Face |
| 5 | Female | 32 | Face |
| 6 | Female | 67 | Face |
| 7 | Female | 82 | Hand |
| 8 | Female | 53 | Neck, face |
| 9 | Female | 71 | Fingernail |
| 10 | Female | 58 | Toenail |
| 11 | Female | 54 | Face |
| 12 | Female | 71 | Back, foot |
